# A Bristol Experience: Benefits and Cost of an "Asthma Nurse"

**Published:** 1989-02

**Authors:** F Carswell, E J Robinson, G Hek, T Shenton

## Abstract

The care of asthmatic children is commonly sub-optimal. Accordingly the costs and benefits of sending a district health authority community nurse, who had specific training in asthma management, into the homes of 43 families with asthmatic children were assessed. During the six months the asthma nurse visited these homes, the children developed and sustained, higher peak expiratory flow rates than 43 control asthmatics. The direct costs of the asthma nurse was £15/patient/six months.


### A Bristol Experience: Benefits and cost of an 'Asthma Nurse' Visiting the homes of asthmatic Children

**F Carswell, MD, PhD, FRCP[1], E J Robinson, PhD[2], G Hek, RGN[3], T Shenton[1]**
[1] Respiratory Research Group, Department of Child Health, University of Bristol
[2] School of Education, University of Bristol
[3] School of Nursing, Bristol Royal Infirmary

### ABSTRACT
The care of asthmatic children is commonly sub-optimal. Accordingly the costs and benefits of sending a district health authority community nurse, who had specific training in asthma management, into the homes of 43 families with asthmatic children were assessed. During the six months the asthma nurse visited these homes, the children developed and sustained, higher peak expiratory flow rates than 43 control asthmatics. The direct costs of the asthma nurse was £15/ patient/six months.

### INTRODUCTION
Diseases such as asthma may have a legitimate case for preferential allocation of new resources, despite their low mortality, because of the large benefit producable at low cost. There is a long-standing failure to deliver the effective remedies for childhood asthma, both when morbidity (1) and mortality (2) are examined. The reported 12% prevalence (3) supports the view that using medically qualified practitioners to directly give the therapy would be too expensive. It would be appropriate to utilise (cheaper) paramedicals in a new service.

Accordingly, the following trial was devised to measure the effectiveness and cost of District Health Authority nurses as providers of support to families with asthmatic children.

### MATERIALS AND METHODS
The study was approved by Bristol and Weston Health Authority Ethical Committee.

Two family group practices were approached and all agreed to participate in the trial. Practitioners not only refrained from finding out the families treatment group but also attempted to prevent the trials occurrence from influencing their asthma management. All families with children aged between 5 and 15 in one suburban and one urban group practice filled out a questionnaire to establish the diagnosis of asthma. This led to the finding of 114 families in whom one child aged 5–15 had asthma; 86 families agreed to participate in the study. The questionnaire responses showed that 73/86 families knew their child had asthma before the trial. The mean age (SD) of these asthmatic children was 11.2 (3.4) years and 59 were boys.

43 of the 86 asthmatic families who agreed to participate were randomly allocated to receive visits by a District Health Authority (DHA) nurse who had been trained for 1 week (whole-time), to have particular skills in the management of asthmatic children. The randomisation was kindly performed by a statistician (Dr Tim Peters). The 'asthma nurses' were full-time community nurses and this project only occupied an average of one tenth of their working week. The nurses visited the homes and discussed asthma, its risks, treatment and factors likely to provoke attacks in detail. The discussion was centred on the affected child's treatment and the nurse indicated appropriate methods of preventing or curtailing attacks. They exercised their professional judgement as to the number of home visits required by each family. The trial was conducted during school term-time, outwith the grass pollen season in the period from 1 October 1985 through to 1 June 1986.

### Indices of potential benefit
The treatment groups' identity was hidden from the nurse investigator (GH) who collected the data from all 86 families and was not involved in the provision of their care.

The highest Peak Expiratory Flow rate (PEF) recorded from 3 properly executed expiratory flow manoeuvres is presented as a percentage of that predicted as normal for the child's height. PEFs were recorded for 1 week periods during the six months of the asthma nurses's intervention. Recordings were made at 8.00 am and 9.00 pm on 7 successive days to produce the mean weekly PEF. Four separate weeks of PEF records were collected at the same times in treated and control subjects: throughout these successive periods, the family also recorded the symptoms which could be ascribed to the child's asthma. All PEF meters (mini-meters, Airmed) were calibrated before and after usage. The coefficient of reproducibility of individual measurements of PEF was 11%.

G H applied structured questionnaires to the child and the parent most involved in the child's asthmatic management to produce a Theoretical Knowledge Score (TKS) which examined this "family unit"'s ability to use objective signs of asthma severity. Four such assessments were applied to all families.

Two-tailed Mann-Whitney tests were used to compare the groups at each of the four assessments.

### RESULTS

### The PEF and Symptoms
The PEF figures in the Table are from those subjects who had a complete PEF data set. The smallest number who had this complete set, was 51 (out of 86) in the fourth assessment of PEF. The apparent direction of change was the same when all the available data was used.

There was a great variation between subjects in the daily symptom scores so that the standard deviations approximated to the mean scores. There were no significant differences between the groups when the symptoms were compared.

**Table**

**Mean PEF (SD) in the Control (C) and Nurse-Treated (T) Groups**

| *Assessment Sequence* | *C* | *T* | *Significance of Difference (p)* |
|---|---|---|---|
| 1 (Baseline) | 99(16) | 99(22) | n.s. |
| 2 | 92(18) | 106(21) | 0.02 |
| 3 | 97(16) | 108(19) | 0.01 |
| 4 | 100(19) | 109(19) | 0.04 |

### Knowledge of asthma

Only the second assessment of theoretical knowledge (TKS) showed a better score in the treated versus the controls ($p<.05$). There was a correlation ($R=0.61$, $p<0.001$) between the number of nurse visits to each family and the change in knowledge score over the six months.

### Cost borne by the Health Service

Two different asthma nurses were employed. One practice population was apparently in a higher socio-economic group than the other. The observed differences between practice results are attributable either to differences in the nurses or to differences in the families. The direct costs of the nurse were calculated from information recorded prospectively by the nurse. These can be summarised as each visit cost £4.30 or that the asthma nurse cost £15/patient/six months. The mean time in the home was 29 minutes.

Data was prospectively collected by the parents. There was no overt differences in drug costs, patient visits to surgery or hospital or family practitioners visits to home. The family groups who were treated by the asthma nurse, were asked at end of the trial if they found her visits helpful. All 43 visited families could identify at least one manifest benefit of the nurse visiting and, although specifically asked, none of them found the visits disruptive or inconvenient. At the conclusion of the trial 24 (18 nurse-treated) out of 86 subjects thought that the peak flow match results could be of value in their personal management of the child.

### Cost borne by families

No differences were detectable in parents' time off work or in the child's school absence (mean 4.1 days/6 months) between the control and treated group asthmatics.

### DISCUSSION

This study has demonstrated objectively that a district health authority (DHA) nurse with special training in the management of childhood asthma, sent into the homes of families with asthmatic children, can produce significant improvement in the child's physical disability as measured by peak expiratory flow rate. The use of practice nurses in a similar way to our nurses should be able to produce the same effect without formal DHA approval being required.

Given that we have demonstrated a significant improvement in the morbidity of our treated asthmatic children at a modest cost, the question remains as to how this improvement was obtained. As the nurse is not permitted to prescribe treatment, but can inform and suggest how to manage the asthma in the home, it appears possible that the mechanism of the nurses' effectiveness was through improving the families' ability to discriminate the severity of attacks and manage their available drugs. There could also be a placebo effect of the nurse.

Others working in the community have commonly reported failure to increase knowledge. Thus only modest improvement was found as a result of a self help programme for asthmatic children in the USA (4). Similarly in England, there is a recently reported failure to produce improvement in physical well being although some increase in knowledge was produced (5). Our nurses visits offered the advantage of a secure (home) site for the family's interview as well as reinforcement of the message on subsequent visits. The nurses were part of the (family-practice-based) community health care and were therefore both more knowledgeable and more acceptable to the families than more specialized nurses. A striking failure is that reported by Mitchell et al (6). These authors randomised 368 paediatric asthmatic patients into intervention and control groups. The families were visited monthly by a community child health nurse for 6 months. The intervention programme seems to have been more theoretically biased than that carried through by our nurses and was assessed some months after discontinuing the programme. One small (total of 26 subjects) trial (7) carried out in North America, found that a nurse educator resulted in fewer hospitalisations, emergency visits, wheezy days and lower costs in the nurse-treated children. They attributed the effects observed to better comprehension and compliance with therapy.

Whatever the mechanism, it seems reasonable to conclude that a home-visiting nurse with specific knowledge of asthma management, can produce objective physical improvement in asthmatic children. Direct costs were £15/child/6 months. Inspection of the table suggests that the major physical benefit had occurred by the second assessment. If that is the case, it is possible the number of nurse visits could be reduced without major reduction in effectiveness.

### Acknowledgements

We gratefully acknowledge the enthusiastic collaboration of Sister J Clutterbuck and S.E.N. Marian Seales and the collaboration of the group practices. We thank Mr David A Hallows, IPFA, for advice on costing; Mr R James, SRN, for his assistance in setting up the study. Allen and Hanbury assisted in the purchase of the peak flow meters.

### REFERENCES

1. SPEIGHT, A. N. P., LEE, D. A. and HEY, E. N. (1983) Underdiagnosis and undertreatment of asthma in childhood. *Brit. Med. J.* **286**, 1253–6.
2. CARSWELL, F. (1985) Thirty Deaths from Asthma. *Arch. dis. Childh.* **60**, 25–28.
3. ANDERSON, H. R., BAILEY, P. A., COOPER, J. S., PALMER, J. C. (1981) Influence of morbidity, illness label and social, family and health service factors on drug treatment of childhood asthma. *Lancet* **ii**, 1030–2.
4. RAKOS, R. F., GRODEK, M. V. and MACK K. K. (1985) The impact of self-administered behavioural intervention program on paediatric asthma. *J. Psychosom Res.* **29**, 101–8.
5. HILTON, S., SIBBALD, B., ANDERSON, H. R., and FREELING, P. (1986) Controlled evaluation of the effects of patient education on asthma in general practice. *Lancet* **i**, 26–29.
6. MITCHELL, E. A., FERGUSON, V. and NORWOOD, M. (1986) Asthma education by community child health nurses. *Arch. Dis. Child.* **61**, 1184–1189.
7. FIREMAN, P., FRIDAY, G. A., GIRA, C., VIERTHALER, W. A. and MICHAELS, L. (1981) Teaching self-management skills to asthmatic children and their parents in an ambulatory care setting. *Paediatrics* **68**, 341–8.

